# A Rare Case of Steroid-Induced Hypothermia in a Patient With Systemic Lupus Erythematosus

**DOI:** 10.7759/cureus.76642

**Published:** 2024-12-30

**Authors:** Jessica Perry, Morgan Streett, Geeth Nadella, Drew Wells

**Affiliations:** 1 Internal Medicine, Methodist University Hospital, Memphis, USA; 2 Pharmacy, Methodist University Hospital, Memphis, USA; 3 Internal Medicine, Methodist Le Bonheur Healthcare, Memphis, USA

**Keywords:** adverse effect, hypothermia, rheumatologic disorder, steroids, systemic lupus erythematosus

## Abstract

A 75-year-old woman with a history of systemic lupus erythematosus (SLE) presented with isolate ocular symptoms, including a left scleral hematoma, elevated erythrocyte sedimentation rate (ESR), and C-reactive protein (CRP). Initial evaluation combined with isolated ocular symptoms raised concerns for giant cell arteritis rather than an SLE flare. Thus, prompt initiation of high-dose intravenous methylprednisolone (250 mg every six hours) was warranted. While on treatment, the patient developed hypothermia (33.4 ^o^C), detected via routine vitals monitoring for hospitalized patients, four days after starting steroids, which resolved spontaneously without intervention. This case reports a rare occurrence of steroid-induced hypothermia in a patient with SLE receiving high-dose glucocorticoids. The underlying mechanisms remain unclear but may involve hypothalamic interference, immune dysregulation, or endothelial dysfunction inherent to SLE, compounded by glucocorticoid-induced antipyretic effects. Unlike previously reported cases, the patient presented with isolated ocular symptoms and received a higher cumulative steroid dose. This case highlights the need for clinicians to recognize hypothermia as a potential adverse effect of high-dose corticosteroids in SLE, even in atypical presentations. Increased awareness, proactive monitoring, and further research into other risk factors that may predispose patients to developing hypothermia are essential to understanding and managing this rare complication.

## Introduction

Hypothermia is defined as a core body temperature of less than 35 degrees Celsius (^o^C) and can have many detrimental clinical complications, including arrhythmias, respiratory failure, altered mental status, coagulation disorders, and metabolic complications [1]. Hypothermia can be pharmacologically induced by agents such as antipsychotics, antidepressants, sedatives, anesthetics, and neuromuscular blockers [1]. Though rare, there have been reported cases of steroid-induced hypothermia, specifically in patients with systemic lupus erythematosus (SLE) [2-8].

SLE is an autoimmune condition in which antibodies attack tissue and organs, including joints, skin, and kidneys, and create a pro-inflammatory state. Mechanisms in which steroids may induce hypothermia in SLE are unknown, but theories include prevention of central recognition of hypothermia [3], impairment of the hypothalamus [7], or the pathology of SLE itself, which may contribute to hypothermia development [4,6,9]. These hypotheses are based on limited case reports and require further investigation. In the reports of steroid-induced hypothermia in SLE patients, patients presented during a lupus flare with clinical features, including fever and confusion. 

Here, we report the case of a 75-year-old female patient with SLE who developed asymptomatic hypothermia after high-dose corticosteroid treatment.

## Case presentation

A 75-year-old woman with a history of SLE, hypertension, and cataracts presented to the emergency department with blurry vision. She was sent by her ophthalmologist after she described worsening vision with dimming and intermittent pain in the left eye over two days. The patient denied slurred speech, numbness, or weakness. Of note, she recently had bilateral cataract surgery, one occurring six weeks prior and the most recent two weeks prior to presentation. Vital signs during admission demonstrated a temperature of 37 ^o^C, a heart rate of 61 beats/min, and a blood pressure of 177/85 mmHg. On examination, the patient was alert, fully oriented, and in no acute distress. The only notable finding was a scleral hematoma in the left eye. Labs were remarkable for an erythrocyte sedimentation rate (ESR) of 109 mm/h (reference range: 0-20 mm/h), C-reactive protein (CRP) of 24.8 mg/L (reference value: < 5 mg/L), and an unremarkable comprehensive metabolic panel and complete blood count. The vascular neurology team initially evaluated the patient due to concern for left retinal artery occlusion. Computed tomography (CT) of the brain, CT angiogram of the head and neck, and magnetic resonance imaging (MRI) of the brain were all unremarkable. Ophthalmology ordered a carotid duplex ultrasound, which showed no acute abnormalities. The differential diagnosis included giant cell arteritis (GCA) due to the visual changes and elevated ESR and CRP. Thus, empiric intravenous methylprednisolone 250 mg every six hours for 12 doses was initiated based on clinical findings until further workup could be completed. A temporal artery biopsy was recommended but was unable to be performed in the hospital. BP was managed with the resumption of home antihypertensives. 

**Figure 1:**
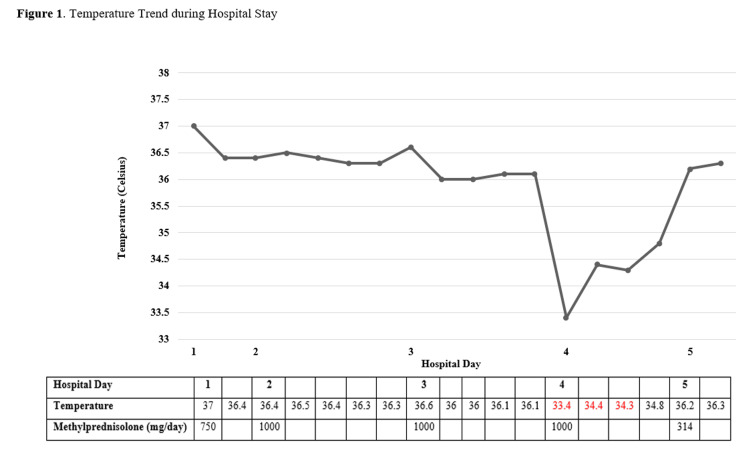
Temperature Trend during Hospital Stay

On the fourth day after the initiation of glucocorticoids, an oral temperature, checked as a part of routine vitals monitoring in hospital patients, of 33.4 ^o^C was recorded. This was confirmed with a rectal temperature of 34.4 ^o^C (Figure 1). The patient’s vision improved while on the steroids and she denied any other symptoms associated with hypothermia. Blood cultures, anti-nuclear antibody (ANA), anti-cardiolipin, and anti-double-stranded DNA studies were collected to rule out other possible causes of hypothermia or alternative autoimmune processes. ANA results were positive with a titer of 1:320 with a homogeneous pattern. The remainder of the workup was negative, including normal thyroid function studies and cortisol level. The patient’s hypothermia resolved without intervention within 48 hours. She was discharged after receiving 4,014 mg of methylprednisolone or methylprednisolone equivalents with plans to continue prednisone 80 mg daily with a taper and schedule an outpatient temporal artery biopsy.

## Discussion

This case highlights the rare consequence of steroid-induced hypothermia in a patient with SLE. While pharmacologic-induced hypothermia is possible, the association with high-dose glucocorticoids is not frequently reported and the mechanism is not well understood [7-9]. This uncommon effect in patients with SLE highlights how susceptible the body’s thermoregulation center is to intrinsic factors and emphasizes the need for awareness of the potential adverse effect of hypothermia in patients with SLE receiving glucocorticoids. The mechanism contributing to hypothermia from steroids in patients with SLE is multi-faceted and controversial. Some studies in vivo and in vitro have demonstrated that glucocorticoids can impair thermoregulation through interference with the hypothalamus, the main source of temperature regulation in the body [8,10]. Other proposed mechanisms include immune system dysregulation and endothelial dysfunction from the inflammatory state created by SLE as well as active antipyretic effects of glucocorticoids that may unmask a predisposition for hypothermia [7,8]. 

There are few cases reported to date of steroid-induced hypothermia in patients with SLE [2,3,6,8]. These cases consisted of patients experiencing SLE flares, receiving high-dose corticosteroids (64-1,500 mg of methylprednisolone equivalents [7-9]), and development of hypothermia within 10-72 hours of glucocorticoid initiation [2-8]. Our patient received high-dose corticosteroids over the five-day hospital course, receiving a total of 4,014 mg of methylprednisolone before discharging on prednisone 80 mg daily in order to provide acute symptom control and mitigate any possible complications or other flares before confirmatory biopsy was obtained. The total amount of glucocorticoids received in our case is significantly greater than previously published cases of steroid-induced hypothermia; however, the high doses are consistent with requirements for autoimmune flares (i.e. GCA, SLE) [7-9]. Our case is also unique in that the hypothermia presented more than 48 hours after the initiation of high-dose steroids, which is counter to previously reported cases [6]. Additionally, other cases included more systemic manifestations of SLE in conjunction with hypothermia, including generalized rash, depressed mentation, dysarthria, and other neuropsychiatric symptoms [3,5,6]. Our patient only displayed ocular symptoms adding to the subtilty of this presentation of a GCA flare versus a mild SLE flare. Thus, it is important to note that hypothermia can occur at any time while receiving high-dose corticosteroids in the presence or absence of additional systemic systems of an SLE flare.

Our case does have limitations, including the presence of active, autoimmune pathologies that could also contribute to hypothermia, the lack of temporal artery biopsy to confirm GCA, and recent cataract surgery that could be on the differential for the inflammatory ocular symptoms. The lack of biopsy does limit the ability to accurately confirm the diagnosis of GCA. However, this does represent a real-world case where clinical certainty is not always established. Thus, a benefit-risk scenario can help guide treatment decisions. In this case, continuation of high-dose steroids proved to be more beneficial to ensure effective resolution of symptoms and prevention of complications. Conversely, our case adds to the limited body of literature that exists describing this phenomenon of hypothermia after high-dose glucocorticoids in patients with SLE. The presentation in this case differs in that it was atypical, constituting only isolated ocular symptoms, and had a unique dose-response relationship in that the hypothermia resolved spontaneously despite the continuation of high-dose corticosteroids.

## Conclusions

This case, with its atypical presentation of isolated ocular symptoms, delayed onset of hypothermia, and higher cumulative steroid dose, contributes to the limited literature on steroid-induced hypothermia in SLE. Further research is needed to elucidate the underlying mechanisms and risk factors, as well as to optimize strategies for prevention and management. For now, clinicians should maintain heightened awareness and include routine temperature monitoring as part of the treatment and monitoring plan for SLE patients receiving high-dose corticosteroids.
